# Transcriptome Responses to Different Environments in Intertidal Zones in the Peanut Worm *Sipunculus nudus*

**DOI:** 10.3390/biology12091182

**Published:** 2023-08-29

**Authors:** Junwei Li, Jiufu Wen, Ruiping Hu, Surui Pei, Ting Li, Binbin Shan, Honghui Huang, Changbo Zhu

**Affiliations:** 1 South China Sea Fisheries Research Institute, Chinese Academy of Fishery Sciences, Guangzhou 510300, China; lijunwei303@163.com (J.L.); nhswjf@163.com (J.W.); liting@scsfri.ac.cn (T.L.); shanbinbin@yeah.net (B.S.); huanghh@scsfri.ac.cn (H.H.); 2 Guangdong Provincial Key Laboratory of Fishery Ecology and Environment, Guangzhou 510300, China; 3 Institute of Biological and Medical Engineering, Guangdong Academy of Science, Guangzhou 510316, China; 4 Corregene Biotechnology Co., Ltd., Beijing 102600, China; peisurui@163.com; 5 College of Animal Science, Inner Mongolia Agricultural University, Hohhot 010018, China

**Keywords:** *Sipunculus nudus*, transcriptome analysis, molecular responses, different expressed genes, different intertidal zones

## Abstract

**Simple Summary:**

*Sipunculus nudus* is a species of significant economic importance because of its high nutritional and medicinal value. It is widely distributed along coastlines worldwide, particularly in China, where a comprehensive industry exists encompassing breeding, farming, processing, and sale. Traditionally, *S. nudus* is cultured on sandy beaches without the need for artificial diets. The organisms rely on nutrients from the surface sediment, including microalgae and other organic matter. These worms inhabit both the intertidal and subtidal zones, enduring varying immersion periods throughout their growth process. As a result, they are exposed to different abiotic factors, such as varying irradiation times, oxygen availability, and others. In our study, we investigated the transcriptomic response of *S. nudus* across high, middle, and low tidal flats. We analyzed and compared the differential gene expression and relevant metabolic pathways in *S. nudus* from different habitats. This analysis aimed to shed light on the molecular mechanisms underpinning physiological responses to diverse environments in burrowing species. Interestingly, we observed that the differential gene expression of *S. nudus* primarily involved metabolic pathways associated with disease and immune responses rather than thermal stress, calcification, and pH regulation. This finding highlights the close relationship between gene expression and *S. nudus’* unique living habits

**Abstract:**

The peanut worm (*Sipunculus nudus*) is an important intertidal species worldwide. Species living in the same aquaculture area might suffer different environmental impacts. To increase knowledge of the molecular mechanisms underlying the response to environmental fluctuations, we performed a transcriptome analysis of *S. nudus* from different intertidal zones using a combination of the SMRT platform and the Illumina sequencing platform. (1) A total of 105,259 unigenes were assembled, and 23,063 unigenes were perfectly annotated. The results of the PacBio Iso-Seq and IIIumina RNA-Seq enriched the genetic database of *S. nudus*. (2) A total of 830 DEGs were detected in *S. nudus* from the different groups. In particular, 33 DEGs had differential expression in the top nine KEGG pathways related to pathogens, protein synthesis, and cellular immune response and signaling. The results indicate that *S. nudus* from different zones experience different environmental stresses. (3) Several DEGs (*HSPA1*, *NFKBIA*, *eEF1A*, etc.) in pathways related to pathogens (influenza A, legionellosis, measles, and toxoplasmosis) had higher expression in groups M and L. *HSPA1* was clearly enriched in most of the pathways, followed by *NFKBIA*. The results show that the peanut worms from the M and L tidal flats might have suffered more severe environmental conditions. (4) Some DEGs (*MKP*, *MRAS,* and *HSPB1*) were upregulated in peanut worms from the H tidal flat, and these DEGs were mainly involved in the MAPK signaling pathway. These results indicate that the MAPK pathway may play a vital role in the immune response of the peanut worm to the effects of different intertidal flats. This study provides a valuable starting point for further studies to elucidate the molecular basis of the response to different environmental stresses in *S. nudus*.

## 1. Introduction

Intertidal zones are special zones affected by the sea and land, with distinctive ecological environments and valuable resources. Therefore, intertidal species must adapt to rapid changes in temperature, desiccation, seawater chemistry (salinity, pH, oxygen, etc.), and other factors. To confront highly dynamic and harsh habitats, intertidal species have evolved different physiological acclimation abilities [1,2]. Thus, intertidal species have become models for investigating the mechanisms underlying tolerance to extreme abiotic conditions [2]. Studies have shown that intertidal species undergo significant physiological changes in response to abiotic factors [3,4]. For instance, shellfish change their energy metabolism, energy stores, cellular stress mechanisms, and cardiac response during environmental fluctuations [5,6,7]. 

However, it is worth noting that biochemical or physiological changes in organisms are caused by gene expression changes under stressful environments [8]. Recent studies have shown that thermal stress induces the differential expression of heat shock protein-related genes in oysters [9]. The genes involved in calcification and pH regulation in sea urchins showed robust changes in response to ocean acidification [10]. Moreover, sea urchin populations from different sites showed the differential expression of genes involved in protein synthesis and biomineralization [11]. Compared to those from a moderate environment, mussels that experienced higher temperatures in rocky intertidal habitats showed the differential expression of genes (*SQSTM1*, *HSPBP1,* and *CRYAA*) that play a role in cellular response to unfolded proteins. Moreover, the description of how gene-expression patterns vary temporally and spatially in the marine rocky intertidal zone was provided [12]. With global climate change causing environmental upheaval, intertidal species have become the focus of a growing body of global change research [2]. Analyzing the molecular response mechanisms of intertidal organisms that underlie tolerance to highly dynamic and harsh habitats may inform predictions about how organisms will respond to global climate change. 

*Sipunculus nudus* is a marine nonsegmented coelomic species found in tidal flats. *S. nudus* has been an important mariculture species in China because of its economic and nutritional value. It is high in amino acids, fatty acids, polysaccharides, and a variety of mineral elements [13,14]. It is known as the “cordyceps of the ocean” in coastal folk culture, and, notably, folk people use the worm for medicinal- and food-conditioning purposes. Modern medical research has demonstrated that the worm contains various active compounds that are useful for enhancing immunity and antifatigue purposes [15,16]. It has been reported that its production can reach approximately 20,000 tons each year in China [17]. Furthermore, it also plays an important role in sustaining ecological balance. It can bury itself down into the sandy sediment to approximately 50 cm, ingest organic matter including microbes from the surface sediment and excrete it in its holes [18], and the depth of ingestion and excretion mainly ranges from 20 to 30 cm [19]. *S. nudus* can utilize the sediment efficiently when present at a high density in the sediment because of their high transport capacity [20]. *S. nudus* is widely distributed along coasts worldwide. Typically, *S. nudus* is cultured along sand beaches without a supplemental artificial diet [17]. However, they can be found in intertidal zones and subtidal zones, where they will endure different immersion days during their growth process. This will affect other abiotic factors, such as different irradiation times, oxygen availability, and other abiotic factors [21]. Furthermore, some better growth characteristics, including stretch and contract ability, and vitality, are observed in high tidal flats. In the present study, we focused on the role of different gene expression patterns and pathways in the ecological adaptation of *S. nudus* to different environments in intertidal zones. We investigated the transcriptomic response of *S. nudus* in high, middle, and low tidal flats and analyzed and compared the differential gene expression and related metabolic pathways in *S. nudus* from different habitats, which is helpful in uncovering the molecular bases of physiological responses to different environments in burrowing species. 

## 2. Materials and Methods

### 2.1. Experimental Animals and Sample Collection

*S. nudus* worms with no apparent injuries or symptoms of disease were collected from 3 sites with different immersion days in a farming zone in the Beibu Gulf, China. The farming area of the peanut worm was more than 1300 ha, and the sampling sites were labeled H (109°48′15” E, 21°21′19” N), M (109°48′17” E, 21°20′58” N), and L (109°48′11” E, 21°20′48” N). The sandy sediment at the H site was soft, and the sediment emerged from the water for 28~30 days each month. The M site had medium–soft sediment, and the sediments emerged from the water for 12~14 days. The L site had a hard bottom with a higher organic content, and the sediments emerged from the water for 4~6 days every month during the low tide. The same batch of juvenile individuals (0.8~1.0 g) of *S. nudus* were cultured in the intertidal flat in March and sampled in November (8~10 g). Three sample plots were designed in parallel in each zone, and 500 g sediment (depth: 0~30 cm) was collected in each plot. The sediment samples were used for the physiochemical analyses, such as the analysis of the salinity in the interstitial water. The total carbon (TOC), total nitrogen (TN), and total sulfur (TS) were measured in the sediment. The sediments in the H, M, and L zones had different environmental characteristics. The final weights of *S. nudus* are shown in Table 1. 

Moreover, the body wall samples of *S. nudus* were collected from the middle third of the body wall and used for the Sirius staining. The treated procedures of the body wall samples included ethanol dehydration and paraffin embedding and sectioning. Then, the dewaxing followed: xylene I for 20 min, xylene II for 20 min, 100% ethanol I for 5 min, 100% ethanol II for 5 min, 75% ethanol for 5 min, and rinsing with tap water. Staining of the section with Sirius Red solution for 8 min, and then dehydration quickly with two or three cups of anhydrous ethanol. Xylene for 5 min, and sealing with neutral gum. The collagen fibers I (crude fiber) would be bright red, and the collagen fibers III (fine fiber) would be green. The area of the collagen fibers were observed using a microscope (Nikon eclipse E100 and Nikon DS-U3, Tokyo, Japan). The ratio of the collagen fibers was based on the histological index.

### 2.2. RNA Extraction, Library Construction, and Sequencing

*S. nudus* were collected from the sample plots in the H, M, and L zones. There were 3 replicates in each group, and a total of 9 individuals were used for the Illumina RNA-Seq and de novo assembly. The transcriptome sequencing and analysis were supported by Annoroad Gene Technology Co., Ltd., Beijing, China. Moreover, in order to obtain a higher quality transcript for reference and comparation, RNA from the 9 individuals were mixed into a sample for PacBio Iso-Sequencing. Body wall samples of *S. nudus* were collected and stored with RNA Safer Reagent, which blocks global transcription of the cells. Prior to the RNA extraction, samples were ground to a powder with a sterilized mortar and pestle in the presence of liquid nitrogen. Total RNA from nine individuals from three treatments was collected for PacBio SMRT (single-molecule real-time sequencing, SMRT) library generation. The RNA was treated with RNase-DNase to remove DNA contaminants. RNA integrity was assessed with an Agilent 2100 Bioanalyzer (Agilent Technologies, Palo Alto, CA, USA). The concentration of each RNA sample was determined using Qubit RNA BR in a Qubit 2.0 Fluorometer (Life Technologies, Carlsbad, CA, USA). For the PacBio Iso-Seq, 1 µg of each RNA sample was pooled together for cDNA library construction. For the Illumina RNA-Seq, an equal amount of total RNA was collected, and indexed cDNA libraries were then prepared for each *S. nudus* sample. The polyA containing mRNA was extracted using oligo-dT attached magnetic beads.

### 2.3. PacBio Iso-Seq Library Preparation and Sequencing

The PacBio Iso-Seq library was prepared according to the Pacific Biosciences protocol. Briefly, 1 µg of polyA mRNA was reverse transcribed into cDNA using the Clontech SMARTer PCR cDNA Synthesis Kit. The first strand cDNA was amplified using KAPA HiFi PCR Kits. The resulting cDNA libraries were purified using 0.40 × AMPure PB Beads, as specified by the supplier (Pacific Biosciences, Menlo Park, CA, USA). Libraries were then size-selected using the BluePippin or Size Selection System (Sage Science, Beverly, MA, USA) into two separate bins each: <4 kb and >4 kb. Each SMRTbell library was constructed using 500 ng size-selected cDNA with the Pacific Biosciences SMRTbell template prep kit 2.1. The binding of SMRTbell templates to polymerases was conducted using the DNA/Polymerase Binding Kit and primers. Sequencing was carried out on the Pacific Bioscience Sequel platform by Annoroad Gene Technology Company (Beijing, China). 

### 2.4. Illumina RNA-Seq and De Novo Assembly

Equal amounts of body wall tissues from the three groups of *S. nudus* were used for RNA extraction. For Illumina RNA sequencing, 3 libraries were obtained in H, M, and L. The RNA was treated with RNase-DNase to remove DNA contaminants, and then the purified RNA quality and quantity were determined using an Agilent 2100RNA Bioanalyzer (Agilent Technologies, Santa Clara, CA, USA). Samples with Qubit BR results (A and B) were used to construct cDNA libraries, and the nine transcriptomic libraries were sequenced on the Illumina HiSeq 2500 platform to obtain 150 bp paired-end reads. For further analysis, the raw reads were filtered by removing the adaptor reads and low-quality reads or unknown nucleotides. The totality of the Illumina clean read mapped to the PacBio isoforms, and the unmapped reads from each library were merged together and then de novo assembled using Trinity Release v24.0 [22].

### 2.5. Transcriptome Assembly, Annotation, and Functional Enrichment

The raw reads underwent quality control (QC) processes, including the removal of linker sequences, adaptors, poly-N sequences, and low-quality reads. The criteria for QC involved eliminating adaptor sequences, retaining polymerase reads with a minimum length of 50 bp, and ensuring a minimum accuracy of 0.8 for polymerase reads. The SMRT Link Portal v9.0 was utilized for QC of the raw reads, followed by analysis using the Iso-Seq3.1.2 pipeline to obtain high-quality, full-length transcripts. To determine Iso-Seq isoform expression, RNA-Seq reads were aligned to the Iso-Seq isoforms derived from Cupcake scripts, using Kallisto. The full-length transcriptome, assembled using PacBio Iso-Sequence, served as the reference genome. Trinity software parameters were as follows: min kmer cov = 4, group pairs distance = 500, and the software was employed for the Illumina short-read assembly, which involved merging and extending the clean reads into longer fragments and transcripts. Subsequently, the merged collection of clean reads was assembled to generate the transcriptome data for *S. nudus*. To explore the biological functions of all unigenes, functional annotation was performed starting with ORF prediction. Complete ORFs with a length exceeding 300 bp were identified, and their corresponding protein sequences were predicted using TransDecoder software (TransDecoder Release v3.0.1). ORF annotation was conducted with Trinotate (v3.0.2) to achieve comprehensive annotation data. For annotation, the unigenes were aligned using BLAST programs against the NCBI nonredundant (NR) protein database, as well as the NT, PFAM, eggNOG, and KEGG databases at an E-value threshold of 1 × 10^−5^. Gene ontology (GO) terms were assigned using Blast2GO [23].

### 2.6. Differentially Expressed Genes and Enrichment Analysis

Mapping reads to the transcriptome assembly: The clean sequencing reads obtained from each of the three libraries (H, M, and L) were aligned back to the assembled transcriptome using Bowtie 2 software with the default parameters [24]. This alignment process allowed us to determine the origin of each read and associate it with specific genes. Identification of DEGs: The H–M, L–H, and M–L groups were compared using the RSEM software [25] to screen for differentially expressed sequences. RSEM was used to align the second-generation high-throughput sequencing data with the full-length transcripts and to quantify the transcripts. By comparing the expression levels of the genes among the different groups, we could identify the genes that showed significant differences in expression using DESeq2. To determine the DEGs, two criteria were applied: an adjusted *p*-value (FDR) threshold of less than 0.01 and a fold-change (FC) threshold of |log_2_(FC)| greater than 1. These criteria were set to define the significance of gene expression differences. The identified DEGs were subjected to functional and pathway enrichment analyses using the Gene Ontology (GO) database and the Kyoto Encyclopedia of Genes and Genomes (KEGG) database. To obtain the background gene set, the assembled full-length transcriptome was annotated using the closely related species database (*Capitella teleta*). After obtaining the background gene set and the gene list file, nonreference genome analysis could be performed by combining the internal function “enricher” of clusterProfiler. These analyses aimed to reveal the biological functions and pathways that were significantly enriched among the DEGs. By comparing the DEGs against known functional categories and pathways, we could gain insights into the potential roles and involvement of these genes in various biological processes. According to the trends in the DEGs’ expression in the H, M, and L regions, the DEGs’ expression in the three groups were clustered using the Mfuzz package in R, utilizing the fuzzy c-means algorithm [26]. This clustering analysis grouped the DEGs into different clusters based on their expression patterns. This helped in identifying subsets of the genes that exhibited similar expression profiles, potentially indicating coordinated regulation and functional relationships. In summary, the analysis involved mapping the reads, identifying DEGs, conducting functional and pathway enrichment analysis, and clustering the DEGs to gain a deeper understanding of the differentially expressed genes and their potential functional implications in *S. nudus.*

### 2.7. Quantitative Real-Time PCR Analysis

Total RNA was extracted from *S. nudus* in three different groups. To confirm the RNA-Seq results (of the selected DEGs), quantitative real-time PCR (qRT–PCR) was performed using AceQ^TM^qPCR SYBR^®^ Green Master Mix (Vazyme, Nanjing, China). Six genes (*CGLTLL*, *HAHM*, *INFKBP*, *PBPAM1*, *AAMTTS*, and *OBNAALAD*2) were selected from the common differentially expressed genes among the three comparisons of the H, M, and L sites for the qRT–PCR validation. Alpha-tubulin was used as an internal reference gene [27]. The primers for the quantitative real-time PCR were designed using Primer Premier 5.0 software, and gene-specific primers were used to detect the relative quantity of each gene. The reaction mixture’s total volume of 20 µL contained 10 µL of 2X SYBR Green PCR Master Mix, 2 µL of cDNA mix, 0.4 µL of each primer, and 7.2 µL of RNase free double-distilled H_2_O. The program for the RT-PCR was 95 °C for 5 min, followed by 45 cycles of 95 °C for 15 s and 60 °C for 60 s. A melting curve analysis was performed at the end of each PCR at 95 °C for 15 s, 60 °C for 60 s, and 95 °C for 15 s, continuously. The relative mRNA levels were calculated using the comparative delta-delta Ct method to normalize and calibrate the gene expression levels relative to the internal reference alpha-tubulin [28]. The genes and primer sequences can be found in Table S1.

## 3. Results

### 3.1. SMRT Sequencing, Illumina HiSeq Sequencing, and Assembly

Each library pool was sequenced independently on six SMRT cells of a Pacific Biosciences Sequel system generating a total of 21.72 Gbp of raw reads. Demultiplexing, stringent filtering, and quality control of the SMRT sequencing data were performed using SMRT Link. The PacBio Iso-Seq sequencing generated 501,988 polymerase reads, and 410,183 high-quality reads with a maximum length of 16.32 kbp and an N50 of 2.67 kbp were generated. The raw polymerase reads from the downstream machine were processed for sequencing junction removal and splitting to obtain the subreads, and 9,717,992 subreads_reads with an average_subreads length of 2192.14 bp and an N50 length of 2379 bp, and mean GC of 0.43 were generated. The full length nonchimeric reads including 5′ primer, 3′ primer, and polyA tail were further clustered into 21,154 consensus isoforms (single representatives of the expressed transcripts) and 21,020 high-quality isoforms with a mean length of 2578 bp. A total of 133 low-quality isoforms were obtained (Table 2). 

We performed Illumina sequencing to evaluate the expression levels of genes in *S. nudus* to understand the molecular characteristics of the organisms in the different intertidal zones. Nine libraries containing three different experimental groups (H, M, and L) were built for the *S. nudus* transcriptome. The Illumina HiSeq sequencing generated 46,464,356 to 63,840,402 raw reads per library. After the trimming of the low-quality reads, final sets of 57,102,456 (H); 60,241,011 (M); and 53,687,205 (L) clean reads were obtained from each pool (Table 3). Among the clean reads, the Q30 percentages in each library were above 93%, which indicates high-sequencing quality. After assembly, the transcriptomes were represented by 105,259 unigenes with a minimum length of 201 bp and a maximum length of 35,077 bp. The transcriptome had an N50 of 1755 bp (Table 2). 

The Benchmarking Universal Single-Copy Orthologs (BUSCOV3.0.1) evaluation resulted in a completeness score of 95.3%. This score was calculated by dividing the sum of complete and single-copy BUSCOs (S, 170) and the complete and duplicated BUSCOs (D, 119) by the total number of BUSCO groups searched (303). 

### 3.2. Expression Levels of Unigenes

**Figure 1:**
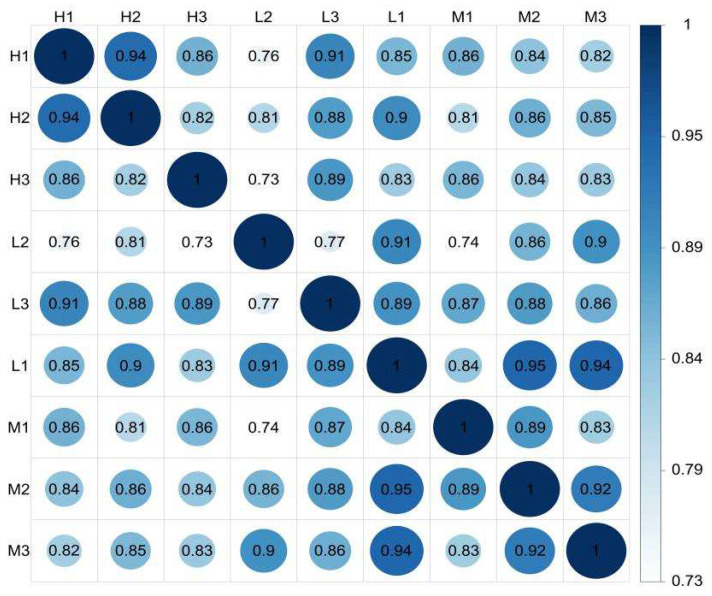
FPKM correlation plot diagram of the expression levels of *S. nudus* from groups H, M, and L (*n* = 3). H: peanut worms from the high tidal flat; M: peanut worms from the middle tidal flat; L: peanut worms from the low tidal flat. The greater the correlation coefficients, the more similar the gene expression model. The correlation coefficients are greater than 0.92 under ideal conditions, and greater than 0.80 is a reasonable value. FPKM: fragments per kilobase of transcript sequence per millions base pairs sequenced.

The FPKM (fragments per kilobase of transcript sequence per millions base pairs sequenced) can be used to analyze the gene expression among different groups [29,30]. The FPKM correlation revealed that *Sipunculus nudus* from the three intertidal zones had the same gene expression profiles, and they belonged to the same species because most of the correlation coefficients were greater than 0.80 (*p* < 0.01). According to the gene expression levels of the unigenes, the correlations of the nine samples were analyzed (Figure 1). The FPKM density distribution is shown in Figure S1. 

### 3.3. Gene Annotation and Function Classification 

**Figure 2:**
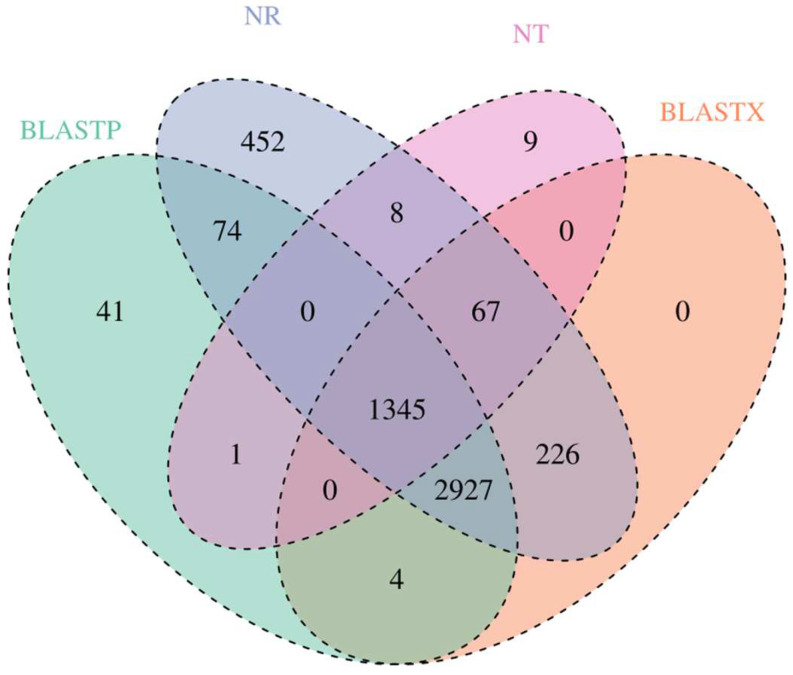
Comparison of the numbers of genes from *S. nudus* annotated to the NR, NT, and UniProt databases. BLASTP represents the number of genes annotated against the UniProt database, and BLASTX represents the number of ORFs annotated against the UniProt database.

The BLAST algorithm was used to realign the high-quality isoforms with open reading frames against the NR, NT, BLASTP, and BLASTX databases. A total of 5099, 1430, 4392, and 4569 annotated unigenes were found in the NR, NT, BLASTP, and BLASTX groups, respectively (Figure 2). To further appraise the completeness of the RNA-Seq data, KOG classifications were performed with the annotated gene sequences, and most of the genes were classified into general function prediction only, signal transduction mechanisms and modification, protein turnover, and chaperones (Figure S2). 

**Figure 3:**
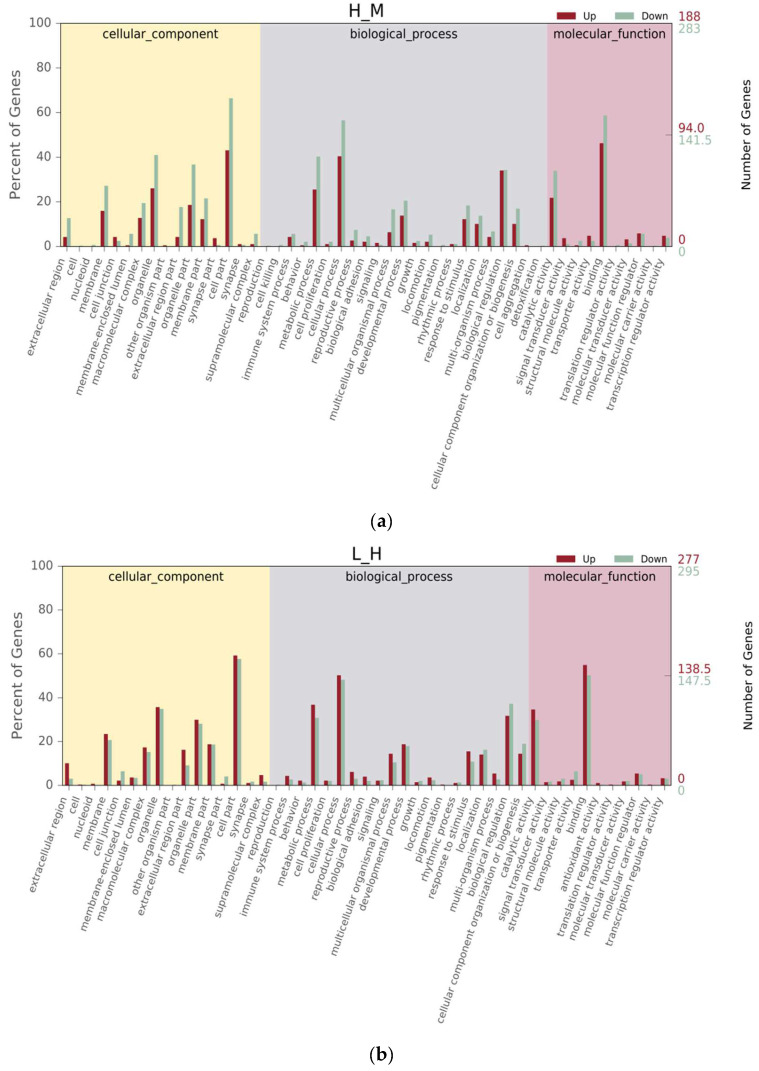

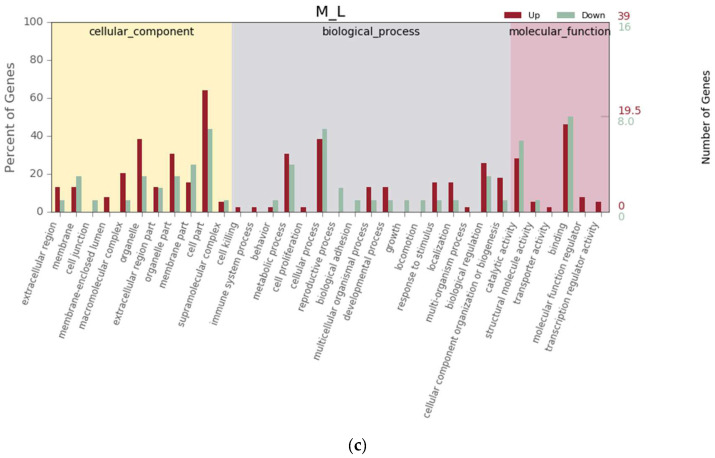
Histogram of Gene Ontology (GO) classification for up-/downregulated unigenes derived from *S. nudus* in the different tidal flats. H: peanut worms from the high tidal flat; M: peanut worms from the middle tidal flat; L: peanut worms from the low tidal flat. (**a**–**c**) Unigenes upregulated or downregulated between H and M, L and H, and M and L, respectively.

Between the H and M groups, GO terms were categorized into 49 subcategories in level 2, with 16 (32.65%) cellular component, 23 (46.94%) biological process, and 10 (20.41%) molecular function (Figure 3a). Between the L and H groups, GO terms were categorized into 47 subcategories in level 2, with 16 (34.04%) cellular component, 20 (42.55%) biological process, and 11 (23.40%) molecular function (Figure 3b). Within the “biological process” category in level 1, the “cellular process”, “metabolic process”, and “biological regulation” were the main subcategories in level 2. Within the “cellular component” category in level 1, the “cell part”, “organelle part”, and “organelle” were the main subcategories in level 2. Within the “molecular function” category in level 1, “binding”, “ion binding”, and “catalytic activity” were the main subcategories in level 2. In this study, 2553 genes, 3088 genes, and 260 genes were successfully matched to the GO database in the comparison groups H vs. M, L vs. H, and M vs. L, respectively. A total of 778 genes were upregulated and 1775 genes were downregulated for H vs. M, 1553 genes were upregulated and 1535 genes were downregulated for L vs. H, and 194 genes were upregulated and 66 genes were downregulated for M vs. L (Figure 3). However, there were no significant differences in the collagen fibers ratio among the groups (*p* > 0.05).

### 3.4. Differential Gene Expression Analysis

**Figure 4:**
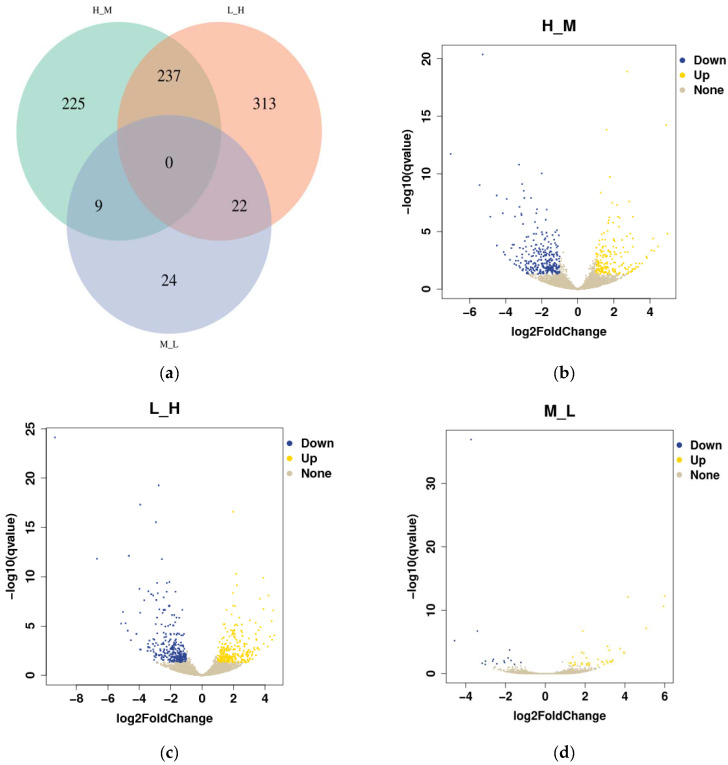
Comparison of the differentially expressed genes (DEGs): (**a**) Venn diagram of overlapping DEGs among the three comparison groups (H vs. M, L vs. H, and M vs. L); (**b**) volcano diagram of DEGs in the comparison group H vs. M; (**c**) volcano diagram of DEGs in comparison group L vs. H; (**d**) volcano diagram of DEGs in the comparison group M vs. L.

In this study, 45,625,705; 46,760,269; and 39,609,198 qualified reads were obtained from the different intertidal zones (H, M, and L). Setting *q* < 0.05 and |log 2 (fold change)| ≥ 1 as the cutoffs, the DEGs annotated in *S. nudus* from the different tidal flats were identified as upregulated or downregulated genes. As shown in the Venn diagram, a total of 830 DEGs were detected in *S. nudus* from the different tidal flats (Figure 4a). Of the DEGs, there were no shared differentially expressed genes among the three groups compared, while 237 genes were differentially expressed in both H vs. M and L vs. H. The number of up- and downregulated genes changed markedly between H and M (188 upregulated vs. 283 downregulated genes), while there was the same gene expression pattern between L and H (277 upregulated vs. 295 downregulated genes); however, there were fewer differential gene expressions between M and L (39 upregulated vs. 16 downregulated genes) (Figure 4b–d).

**Figure 5:**
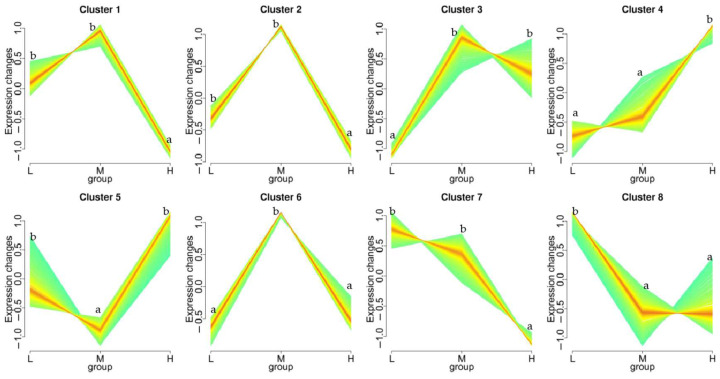
Trend analysis of DEGs in *Sipunculus nudus* responding to different tidal flats. Eight significant clusters are displayed as tidal flat plots of expression ratios. The number of genes assigned to each cluster is shown. The expression trend analysis is based on the resulting datasets identified as eight clusters of genes with characteristic transcription response profiles. Different lowercase letters (a, b) represent significant differences among the treatments (*p* < 0.05, Wilcoxon test).

The trend analysis results show that the DEGs were grouped into eight gene expression patterns (Figure 5). In cluster 1 (78 genes) and 2 (15 genes), the DEGs were upregulated in groups L and M compared to group H, respectively. The DEGs were mainly related to tRNA ligase, elongation factor, heat shock protein 70, stress-70 protein, tubulin alpha-1 chain, and some hypothetical proteins that have been found in *Capitella teleta*. Moreover, there was an expression pattern with a significantly upregulated expression trend (cluster 7, 33 genes) with an increasing soaking time; however, there was no significant difference between the land M groups. The DEGs in cluster 7 were mainly related to the protein skeleton, low-density lipoprotein receptor, cytochrome, signal peptide, aldehyde dehydrogenase, and some hypothetical proteins (*C. teleta*). Cluster 4 (82 genes) was significantly upregulated in terms of an expression trend in groups L, M, and H with exposure time. The DEGs were mainly related to ubiquitin-protein ligase, galactosylceramide sulfotransferase, serine/threonine-protein phosphatase regulatory ankyrin, zinc finger protein, heat shock protein beta-1, Ras-related protein, dual-specificity MAP kinase phosphatase, AMP-activated protein kinase, and some hypothetical protein (*C. teleta*). 

### 3.5. KEGG Pathway Enrichment Analysis of the Genes

**Figure 6:**
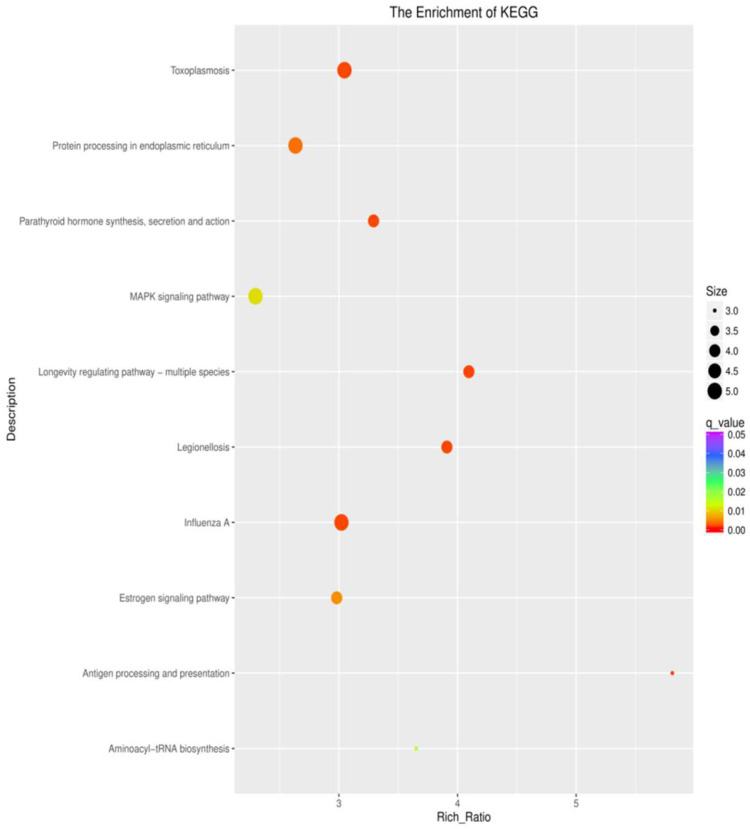
KEGG enrichment analysis of the DEGs in *S. nudus.* The area of each colored circle is proportional to the number of genes involved in each pathway, the color indicates the q-value, and the x-axis is the Rich ratio.

To understand the regulation of the gene expression in response to the different intertidal zones, the DEGs were compared against the KEGG database for pathway enrichment in the comparison groups of H vs. M, L vs. H, and M vs. L. A total of 324 pathways were mapped. There were 25, 17, and 2 enriched pathways in the different comparison groups (H vs. M, L vs. H, and M vs. L) (*p* < 0.05), respectively. A number of immunity-related pathways were enriched in the H vs. M and L vs. H groups, such as pathways related to pathogens and protein synthesis, including legionellosis, influenza A, measles, toxoplasmosis, longevity-regulating pathway—multiple species, and aminocayl-tRNA biosynthesis. Pathways related to the cellular immune response and signaling, including antigen processing and presentation, and the MAPK signaling pathway were enriched in the H vs. M and L vs. H groups. Pathways related to hormones, including the estrogen pathway and oxytocin signaling pathway, were enriched in the H vs. M and L vs. H groups (Figure 6; Table S2). However, the above pathways were not enriched in the comparison group of M vs. L. 

The DEGs in cluster 1 were mainly enriched in KEGG pathways related to disease, chemical damage, and immune responses (influenza A, legionellosis, antigen processing and presentation, aminocayl-tRNA biosynthesis, FoxO signaling pathway, longevity-regulating pathway—worm, protein processing in the endoplasmic reticulum, hippo signaling pathway, pathways in cancer, AMPK signaling pathway, RNA transport, tight junction, melanogenesis, RNA transport, and chemical carcinogenesis). The DEGs in cluster 2 were mainly enriched in KEGG pathways related to disease and immune responses (influenza A, legionellosis, antigen processing and presentation, measles, MAPK signaling pathway, FoxO signaling pathway, melanoma, toxoplasmosis, linoleic acid metabolism, estrogen signaling pathway, longevity-regulating pathway—multiple species, pathogenic Escherichia coli infection, Toll and Imd signaling pathway). The DEGs in cluster 7 were also mainly enriched in KEGG pathways related to disease and immune responses (legionellosis, influenza A, measles, toxoplasmosis, aminocayl-tRNA biosynthesis, Toll and Imd signaling pathway, NOD-like receptor signaling, and chemokine signaling pathway). However, the DEGs in cluster 4 were mainly enriched in KEGG pathways related to cell proliferation, differentiation and migration (MAPK signaling pathway, pathways in cancer, hippo signaling pathway, FoxO signaling pathway, chemokine signaling pathway, apelin signaling pathway, oxytocin signaling pathway, tight junction, bladder cancer, AMPK signaling pathway, linoleic acid metabolism, nonsmall cell lung cancer, and longevity-regulating pathway). The DEGs and function annotations are presented in Figure S4.

The legionellosis pathway was characterized by the presence of four differentially expressed genes (DEGs), namely, *HSPA1*, *NFKBIA*, *eEF1A*, and *GroEL*. These DEGs were found to be upregulated in both the M and L groups compared to the H group. In the influenza A pathway, the M and L groups exhibited the upregulation of seven DEGs (*HSPA1, NFKBIA, UAP56, PRSS, MEK1, EIF2S1,* and *FURIN*) and four DEGs (*HSPA1, NFKBIA, UAP56,* and *PRSS*), respectively. Regarding the measles pathway, the M group showed significant upregulation of three genes (*HSPA1, NFKBIA,* and *EIF2S1*), while the L group displayed upregulation of two genes (*HSPA1* and *NFKBIA*). Similarly, in the toxoplasmosis pathway, the M and L groups exhibited upregulation of two genes (*HSPA1* and *NFKBIA*), and one DEG (*MAP3K7IP1*) was found to be downregulated in the M tidal flat. In the longevity-regulating pathway—multiple species pathway, the M and L groups demonstrated upregulation of two DEGs (*HSPA1* and *HSP20*) and two DEGs (*HSPA1, SODC_PBCV1*), respectively. Additionally, the M and L groups displayed downregulation of two DEGs (*PRKAA* and *PRKAB*) and three DEGs (*PRKAA* and *AKTS1_HUMAN*), respectively. In the aminoacyl-tRNA biosynthesis pathway, the M and L groups showed significant upregulation of six genes (*QARS, TARS, SARS, RARS, KARS,* and *DARS*) and five genes (*QARS, TARS, SARS, RARS,* and *KARS*) associated with tRNA synthetases, respectively. The antigen-processing and -presentation pathway exhibited significant upregulation of three genes (*HSPA1*, *CANX*, and *HtpG*) in both the M and L groups. In the MAPK signaling pathway, the M and L groups displayed significant upregulation of three genes (*HSPA1, GADD45,* and *MEK1*) and three genes (*HSPA1, STMN1_CHICK,* and *ANGP4_BOVIN*), respectively. Additionally, several DEGs (*HSPB1, MKP,* and *MRAS*) were significantly downregulated in the M group, while the L group showed downregulation of HSPB1, MKP, and MRAS. Within the estrogen pathway, the M group exhibited significant upregulation of four DEGs (*HSPA1, HSP90A, MAP2K1,* and *FKBP4_5*), and the L group showed upregulation of four DEGs (*HSPA1, HSP90A, FKBP4_5,* and *CALL4_BOVIN*). In the oxytocin signaling pathway, two genes (RHOA and MEK1) were significantly upregulated in the M group, while the L group showed upregulation of *RHOA* and *CALL4_BOVIN*. Several DEGs (*PRKAG, PRKAB, EEF2K,* and *CAMK2*) were significantly downregulated in the M group, and in the L group, the downregulated DEGs included *PRKAG, EEF2K, CAMK2, CALM_CIOIN, RYR1_RAT,* and *ADCY9_MOUSE* (Table S2). Among the DEGs, *HSPA1, MEK1, NFKBIA, GADD45, HSP90A,* and *CANX*, which belonged to clusters 1, 2, and 7, appeared most frequently in the comparison groups (H vs. M, M vs. L, and L vs. H). These DEGs are presented in Table S3.

### 3.6. Validation of the Transcriptome with qRT–PCR

**Figure 7:**
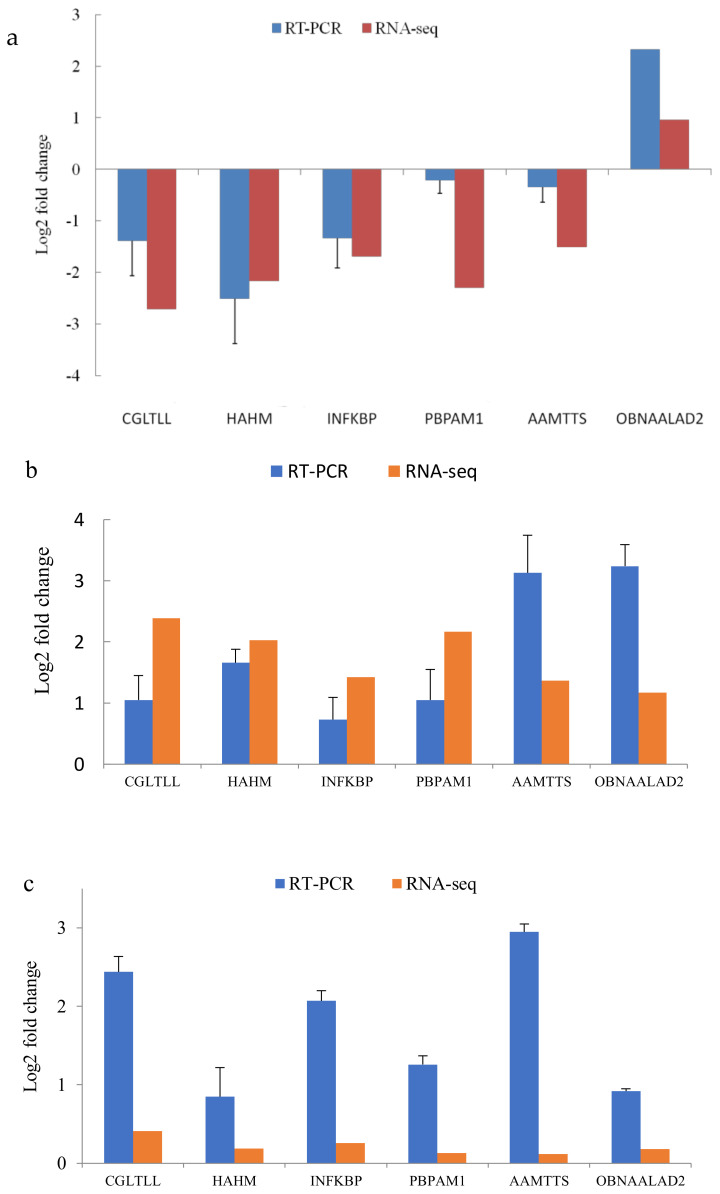
Comparison of the relative fold changes between the qRT-PCR and RNA-Seq results for the following groups: (**a**) H vs. M; (**b**) M vs. L; (**c**) L vs. H. The expression levels of the selected genes were normalized to that of the α-tubulin gene. *CGLTLL*: Crassostrea gigas lysine-tRNA ligase-like; *HAHM*: Haliotis asinina Hsp90A mRNA; *INFKBP*: inhibitor of nuclear factor-kappaB protein; *PBPAM1*: Python bivittatus protein arginine methyltransferase 1; *AAMTTS*: Apteryx australis mantelli threonyl-tRNA synthetase; *OBNAALAD*2: Octopus bimaculoides N-acetylated-alpha-linked acidic dipeptidase 2-like.

To verify the Illumina sequencing results, six genes were selected at random for further confirmation using qRT–PCR. In the selection process of these genes, the main aim was to verify the validity of the results. As shown in Figure 7, the verification results indicate that there was no deviation in the expression patterns among methods, as all tested genes were consistent with the data obtained from the transcriptome analysis with only slight variations in the expression levels.

## 4. Discussion

### 4.1. Transcriptomic Characteristics of Sipunculus nudus in Tidal Flats

*Sipunculus nudus*, similar to other intertidal organisms, lives in an environment that alternates between aquatic and terrestrial due to the rise and fall of the tide [17]. Moreover, peanut worms are widely distributed in intertidal flats, and they endure different habitats and different cycles of immersion and emersion during their growth process. Thus, there might be some changes at the genetic level in *S. nudus* from different intertidal environments. In the present study, the DEGs and KEGG pathways related to the immunity and growth of *S. nudus* from different intertidal zones were first analyzed using a combination of the SMRT platform of Pacific Biosciences and Illumina RNA-Seq. In total, we found 830 DEGs (upregulated genes and downregulated genes) in the body wall of *S. nudus* from different intertidal zones. The DEGs were further subjected to KEGG pathway enrichment analysis to assist in understanding the potential molecular mechanisms that underlie the response to different intertidal zones. Because of the similar environments of the M and L tidal flats, as well as the fewer DEGs found between the M and L groups, the results of the comparison groups of H vs. M and L vs. H were mainly analyzed. In the GO analysis, the DEGs were mainly involved in cell-related categories, which suggests that *S. nudus* from the M and L groups underwent relatively strong changes to enhance cell function and resist environmental changes. In the KEGG analysis, several pathways related to pathogens, protein synthesis, and cellular immunity were enriched in the comparison groups H vs. M and L vs. H (*p* < 0.05). Other studies have indicated that pathways related to influenza A, legionellosis, and other pathways related to disease or immunity in fish and shrimp could be induced by pathogens or heavy metals [31,32,33,34]. Influenza A, legionellosis, and measles are related to immune and infectious diseases, and these pathways can be activated by pathogen infections under acute thermal stress [35]. The above pathways were enriched in the present study, which indicates that these pathways might play an important role in the response of *S. nudus* to the different intertidal environments. However, enriched pathways related to muscle contraction were not observed in the present study. Moreover, there was no significant difference in the ratio of collagen fibers III among the three groups (Figure S3).

A previous study also showed that pathways related to legionellosis and influenza A were enriched in the larval period of *S. nudus* compared to the pelagospheric period [36]. Moreover, the enriched KEGG pathways, such as influenza A and measles, were found in *R. philippinarum* but not in *Crassostrea gigas*, *Pinctada fucata*, or *Patinopecten yessoensis* from their growth environment [37]. Perhaps shellfish living in sediment are more susceptible to the effects of pathogens than attached shellfish. Previous studies showed that there were multiple species of pathogens in estuarine or coastal sediment [38,39]. Sediments with greater organic matter content and lower porosity harbored greater numbers of nonculturable bacteria, and the abundance of bacteria confirms the role of sediments in the accumulation and persistence of fecal bacteria [40]. In the present study, the differentially expressed genes (DEGs) associated with disease-related pathways, such as influenza A, legionellosis, measles, and toxoplasmosis, exhibited upregulation in the M and L intertidal zones, and higher organic matter (Table 1) and lower light occurred in the sediments of the M and L zones than that in the H zone. The environmental situation may be responsible for the higher relative abundance of pathogens in the M and L zones. 

Similar to other invertebrates, *S. nudus* depends on innate immune responses to deal with environmental stressors. MAPK pathways are highly conserved across animal taxa and occupy a central position in various fundamental biological processes [41,42], and these pathways are considered to play a vital role in the worm immune response because of their function in regulating bacterial infections, stress responses, and longevity [43]. In addition, the MAPK pathway can control germline aging, and it can be used to attenuate the rate of the decline in oogenesis quality [44]. In the present study, the *HSPA1* gene in the MAPK signaling pathway was significantly upregulated in both the M and L groups. In addition, several DEGs (*HSPB1*, *MKP*, and *MRAS*) were downregulated in the M and L groups compared to group H. These results indicate that *S. nudus* may increase immune system activity by enhancing the MAPK signaling pathway in response to the threat of pathogens or the environment in the tidal flat. Another significantly enriched pathway is aminoacyl-tRNA synthesis; its function is to precisely match amino acids with the tRNA containing the corresponding anticodon [45]. A previous study found that the aminoacyl-tRNA biosynthesis pathway in gastric tissue was markedly upregulated in the pathogenic state [46]. In the present study, the DEGs upregulated in this pathway in the M and L groups indicate that *S. nudus* may increase physiological activity by enhancing the aminoacyl-tRNA synthesis pathway during the immune response.

### 4.2. Analysis of Differentially Expressed Genes in KEGG Pathways

Several genes (*HSPA1*, *MEK1*, *NFKBIA*, *GADD45*, *HSP90A*, and *CANX*) assigned to clusters 1, 2, and 7 appeared most frequently in the comparison groups. In the present study, *HSPA1* was upregulated in the M and L groups compared to the H group, and it was differentially expressed in most of the top nine enriched pathways. *HSPA1* belongs to the *Hsp70* protein family, which has often been reported to be relevant for resisting harmful environments [32], and the expression level of *HSPA1* increases when aquatic animals are subjected to bacterial infection, parasites (*C. irritans*), thermal stress, and heavy metal toxicity [31,33,34]. The expression level of *Hsp70*-binding protein (*HSPBP1*) increases when mussels experience higher temperatures in rocky intertidal habitats [12]. This study demonstrated that *HSPA1* is sensitive in *S. nudus* and can be used to predict the conditions of the habitat or the survival state. The overexpression of *HSPA1* showed that *S. nudus* living in the M and L tidal flats was exposed to more severe environments than *S. nudus* living in the H tidal flat. Similar results were found for the *NFKBIA* gene, which was enriched in the influenza A, legionellosis, measles, and toxoplasmosis pathways; moreover, it was also upregulated in the M and L groups compared with the H group. The *NFKBIA* gene encodes one piece (the alpha subunit) of the *IKK* protein complex, which is considered to correlate with the inflammation responding to *Plesiomonas shigelloides* in the southern catfish [47]. The gene was also found in connection with the pathogenesis of human cancer [48]. We speculate that the *NFKBIA* gene might be an important gene for the *S. nudus* response to severe environmental conditions or pathogen infection. Eukaryotic translation elongation factor 1A (*eEF1A*) is one of the most abundant protein synthesis factors. *eEF1A* is responsible for the delivery of all aminoacyl-tRNAs to the ribosome, except for initiator and selenocysteine tRNAs [49]. It has a well-defined role in protein synthesis, and it regulates HSP70 expression and thermotolerance [50]. Overexpression of *eEF1A* has been proposed to be related to cell proliferation and cancer development [51]. In the present study, *eEF1A* was upregulated in peanut worms from the M and L tidal flats. The results indicate that the worms might produce more protein in response to sedimentary environments that have lower porosity and greater organic matter or even a high abundance of pathogens. *MEK1* and *GADD* are essential for the regulation of proliferation and apoptosis; *MEK1* is activated by various environmental stresses and proinflammatory cytokines [52], and *GADD* is induced by various stimuli, including DNA damage and ER stress [53]. In the present study, *MEK1* and *GADD* were upregulated in the M group compared to the H group, although they participated in different pathways. *MEK1* was enriched in the influenza A, MAPK, FoxO signaling pathways, as well as the estrogen signaling pathway, and *GADD* was enriched in the MAPK and FoxO signaling pathways. According to the expression trends, the DEGs assigned to clusters 1, 2, and 7 were upregulated, and the DEGs responded to the unsuitable environment in the M and L tidal flats. In addition, some DEGs (*PRSS*, *UAP56*, *FURIN*, and *EIF2S1*) showed similar trends and were enriched in the influenza A pathway, and they were upregulated in the M tidal flat compared to the H tidal flat. The results indicate that *S. nudus* living in the M tidal flat might suffer from poor environmental conditions or exposure to pathogens. Furthermore, the higher abundances of legionellosis, *Escherichia coli* shigella, and *Stenotrophomonas* were found in the gut of *S. nudus* from the same tidal flat [54]. The tidal flat might be contaminated to some extent, and the gut provided more suitable conditions for these pathogens, and there might already be adaptation between *S. nudus* and pathogens.

Most of the DEGs were upregulated in the M or L tidal flat. However, some DEGs (*MKP*, *MRAS*, and *HSPB1*) were upregulated in *S. nudus* from the H tidal flat, and these DEGs were mainly differentially expressed in the MAPK signaling pathway. *MKP* (dual-specificity *MAP* kinase phosphatase) provides a complex negative regulatory network that shapes the duration, magnitude, and spatiotemporal profile of MAP kinase activities in response to both physiological and pathological stimuli [55]. Reactive oxygen species (ROS) can kill or inhibit biological cells [56]; moreover, ROS in moderation can improve the expression level of *MKP*-*1* [57]. We speculated that *S. nudus* and the sediment from the H tidal flat were exposed to much more ROS than those in the M and L tidal flats because of prolonged exposure to ultraviolet rays. In the present study, the sandy sediment in the H zone was soft with good permeability, and the sediment had a longer exposure period in the open air. The above factors can increase the ROS in the H tidal flat. According to our findings, enhancing the soil porosity or utilizing more ROS may improve the physiological state of *S. nudus*, consequently boosting its growth performance.

## 5. Conclusions

The worms from the different tidal flat zones have different molecular bases of physiological responses to the different environmental conditions. The DEGs of *S. nudus* were mainly enriched in the KEGG pathways related to disease and immune responses rather than thermal stress, calcification, and pH regulation, which is closely related to their special living habitat. Because of the potential contamination from the sediments and the differences in the sediments itself, the DEGs related to tides and, consequently, the environmental consequences could not be accurately inferred. However, worms living in sediments (M and L) with lower porosity and greater organic matter content might encounter even more severe conditions than those in the high tidal flats (H) although situated within the same farming zone.

## Figures and Tables

**Table 1:** Environmental parameters of the sediment and body weight of *S. nudus* in the different tidal flats.

	TN (%)	TOC (%)	TS (%)	Salinity (‰)	Body Weight (g)
H	0.002 ± 0.001	0.0586 ± 0.005	0.600 ± 0.211	24 ± 0.3	9.8 ± 1.4
M	0.004 ± 0.001	0.099 ± 0.0028	0.335 ± 0.073	23 ± 0.2	8.6 ± 1.3
L	0.011 ± 0.001	0.2559 ± 0.049	0.337 ± 0.105	23 ± 0.5	7.8 ± 1.3

TN: total nitrogen; TOC: total organic carbon; TS: total sulfur.

**Table 2:** Summary of the transcriptome of *S. nudus* using PacBio Iso-Seq and Illumina RNA-Seq.

Parameters	PacBio Iso-Seq	Illumina RNA-Seq
Sequencing data		
Number of subreads or raw reads	9,717,992	58,212,575
Number of CCS or clean reads	410,183	57,010,224
Full-length or assembled transcriptome		
Number of transcripts	21,154	105,259
Number of nucleotide bases (Mb)	54.67	
GC content (%)	43.83	39.44
Mean length	2584.71	924.00
Minimum length (bp)	209	201
Maximum length (bp)	11,923	35,077
N50	2673	1755
Length range of transcripts (bp)		
<400	108	47,105
400–1000	674	30,851
1000–2000	5835	14,791
2000–3000	8722	6451
>3000	5815	6061

**Table 3:** Assembled quality of sequenced *Sipunculus nudus* transcriptomes.

Sample	Raw Reads	Clean Reads	Low-Quality Reads Rate (%)	Clean Q30 Bases Rate
H1	58,231,232	57,068,540	0.52	93.97
H2	56,276,300	55,083,032	0.5	94.19
H3	60,401,216	59,155,796	0.63	93.88
M1	63,840,402	62,436,042	0.87	93.09
M2	59,975,242	58,780,862	0.6	93.94
M3	60,770,110	59,506,128	0.52	94.01
L1	46,464,356	45,751,916	0.57	94.88
L2	54,854,172	53,396,614	0.52	94.43
L3	63,100,148	61,913,086	0.5	94.19

H: peanut worms from the high tidal flat; M: peanut worms from the middle tidal flat; L: peanut worms from the low tidal flat.

## Data Availability

Raw sequence data were submitted to the NCBI Sequence Read Archive with accession number: PRJNA916931.

## Supplementary Materials

The following supporting information can be downloaded at: https://www.mdpi.com/article/10.3390/biology12091182/s1, Figure S1: Violin diagram of the expression quantity of unigenes of *S. nudus* from groups H, M, and L (*n* = 3); Figure S2: KOG function classification of transcripts. Figure S3: Sirius staining of the musscle of *S. nudus*. Table S1: Names and primer sequences of genes used for quantitative real-time PCR analysis. Table S2: The enriched KEGG pathways and DEGs in the H vs. M and L vs. H groups. Table S3: Table S3. The annotated genes in the top 10 pathways from KEGG enrichment analysis. Table S4: The DEGs and function annotation present in clusters 1, 2 and 7.
