# Supplementary material for: Transcriptome Responses to Different Environments in Intertidal Zones in the Peanut Worm Sipunculus nudus
Source: Biology (Basel). 2023 Aug 29;12(9):1182. doi: 10.3390/biology12091182 (PMC10525638; doi:10.3390/biology12091182)
Supplement: Supplementary file 1 [file biology-12-01182-s001.zip › biology-2396441-supplementary.pdf]

biology

Supplementary Materials

Transcriptome responses to different environments in intertidal zones in the peanut worm *Sipunculus nudus*

Junwei Li [1,2]*, Jiufu Wen [1,2], Ruiping Hu [3]*, Surui Pei [4], Ting Li [1,2], Binbin Shan [1,2], Honghui Huang [1,2] and Changbo Zhu [5]*

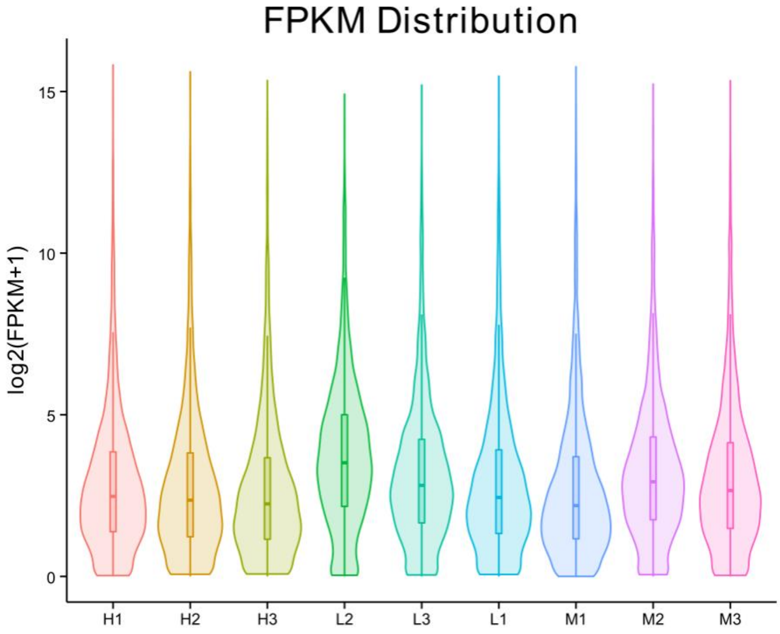

Figure S1. Violin diagram of the expression quantity of unigenes of *S. nudus* from groups H, M, and L (n=3). H represents the peanut worms from the high tidal flat, M represents peanut worms from the middle tidal flat, and L represents the peanut worms from the low tidal flat. FPKM (Fragments Per Kilobase of transcript sequence per Millions base pairs sequenced).

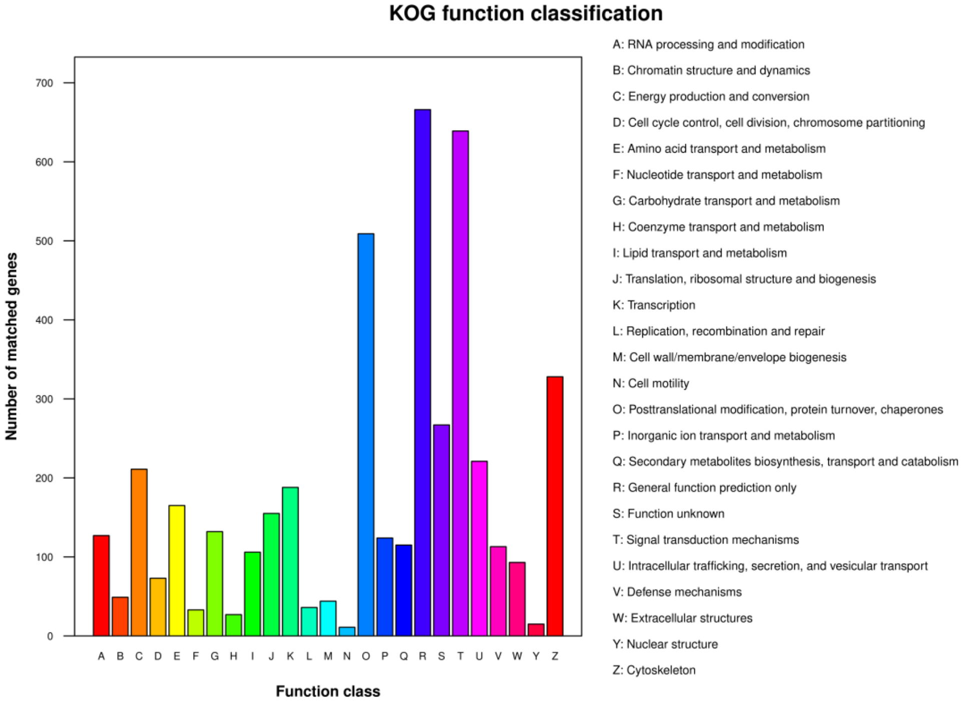

Figure S2. KOG function classification of transcripts. The 23063 unigenes were grouped into 25 KOG categories.

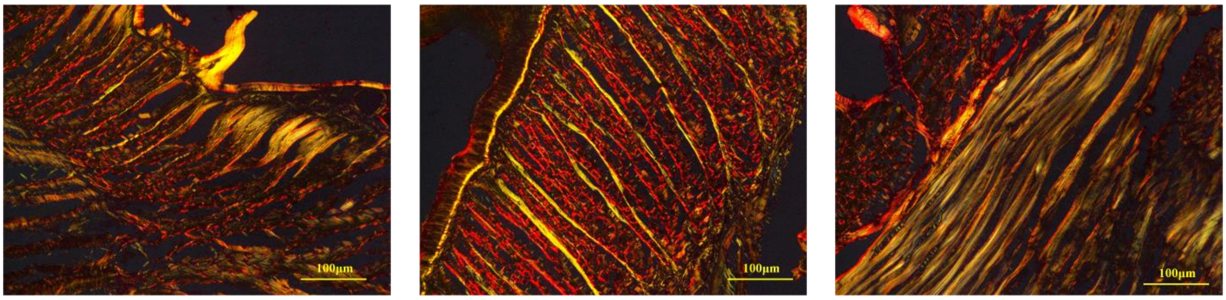

a b c

Figure S3. Sirius staining of the musscle of *S. nudus*. The bright red and crude fiber is collagen fiber I, the green and fine fiber is collagen fiber III. a means the sirius staining of *S. nudus* from H site, b means the sirius staining of *S. nudus* from M site, c means the sirius staining of *S. nudus* from L site.

Table S1. Names and primer sequences of genes used for quantitative real-time PCR analysis.

**Gene name**	Primer sequence		length
**alpha-tubulin**	F	GTGATTTGGCCAAGGTGCAG	141bp
	R	TACCCTCTCCCACGTACCAG	
Crassostrea gigas lysine--tRNA ligase-like	F	CGCTTGAGTATGGTTTGCCG	163bp
	R	GCTGCATGTTGTTCAGCTCC	
Haliotis asinina Hsp90A mRNA	F	TGGAAGGACAGCTTGAGTTCAG	181bp
	R	GAATCCACCACACCCTTGATGA	
inhibitor of nuclear factor-kappaB protein	F	TGATGCCCAGGAGGGAAAAC	124bp
	R	ATCGTAAGACCGTGCGTGAA	

Python bivittatus protein arginine methyltransferase 1	F	CGTTCTCAGCACCATTTCACC	204bp
	R	TTGGCCAAATAGCTCCTCTCC	
Astoria latipes glutaminyl-tRNA synthetase	F	CAGGTTACGTGGTACGGGTG	103bp
	R	TTAGGTTTTGGTGCCTCGGT	
Apteryx australis mantelli threonyl-tRNA synthetase	F	CAGGTTACGTGGTACGGGTG	113bp
	R	TTAGGTTTTGGTGCCTCGGT	
Octopus bimaculoides N-acetylated-alpha-linked acidic dipeptidase 2-like	F	ATGCAGGTCTCACGAGCATTT	108bp
	R	AGCGCTGTTTGTTGAACTTGG	

Table S2. The enriched KEGG pathways and DEGs in the H vs. M and L vs. H groups.

ID		H-M					L-H			
**Pathway name**	**Description**	**Gene**	**Change**	**Fold**	**P-value**	FDR	Change	Fold	P-value	FDR
MAPK	Sipunculus_f7p60_2477_transcript9537_g1	HSPA1	down	-1.81	4.08E-07	4.00E-05	up	3.41	4.61E-08	4.80E-06
	Sipunculus_f2p56_1933_transcript15246_g1	GADD45	down	-1.91	0.000737	0.013193				
	Sipunculus_f5p60_3036_transcript5531_g1	MEK1	down	-1.39	1.82E-07	2.03E-05				
	Sipunculus_f2p30_2444_transcript9857_g1	HSPB1	up	3.05	4.06E-07	4.00E-05	down	-2.44	1.89E-05	0.00072
	Sipunculus_f2p39_1965_transcript14895_g1	MKP	up	1.61	4.29E-05	1.44E-03	down	-1.57	0.000138	0.00333
	Sipunculus_f2p39_2685_transcript7898_g1	MRAS	up	1.91	9.86E-11	3.23E-08	down	-2.24	0.000102	0.002607
	Sipunculus_f2p60_2253_transcript11804_g3	MKP	up	1.51	2.27E-03	2.95E-02	down	-1.62	0.002024	0.025167
Influenza A	Sipunculus_f2p28_3135_transcript5416_g1	NFKBIA	down	-1.69	0.00097914	0.0161544	up	1.42	0.001698	0.022183
	Sipunculus_f2p34_1933_transcript15070_g1	UAP56	down	-1.35	3.28E-05	0.00117238	up	1.53	0.00035	0.006924
	Sipunculus_f4p60_1582_transcript18224_g1	PRSS; trypsin	down	-1.79	1.50E-05	0.00065807	up	1.73	0.000768	0.012306
	Sipunculus_f5p60_3036_transcript5531_g1	MEK1	down	-1.39	1.82E-07	2.03E-05				
	Sipunculus_f6p60_3095_transcript5232_g1	EIF2S1	down	-1.58	0.00095804	0.01594029				
	Sipunculus_f7p60_2477_transcript9537_g1	HSPA1	down	-1.81	4.08E-07	4.00E-05	up	3.41	4.61E-08	4.80E-06
	Sipunculus_f8p60_3918_transcript2145_g1	FURIN, PCSK3	down	-2.01	0.00025075	0.00594689				

Legionellosis	Sipunculus_f11p60_2784_transcript6547_g1	groEL, HSPD1	down	-1.55	0.00019876	0.00509005	up	1.53	8.34E-05	0.002263
	Sipunculus_f2p28_3135_transcript5416_g1	NFKBIA	down	-1.69	0.00097914	0.0161544	up	1.42	0.001698	0.022183
	Sipunculus_f6p60_1939_transcript15139_g2	EEF1A	down	-4.48	2.33E-06	0.00016503	up	4.17	3.52E-05	0.001185
	Sipunculus_f7p60_2477_transcript9537_g1	HSPA1	down	-1.81	4.08E-07	4.00E-05	up	3.41	4.61E-08	4.80E-06
Antigen processing and presentation	Sipunculus_f7p60_2477_transcript9537_g1	HSPA1	down	-1.81	4.08E-07	4.00E-05	up	3.41	4.61E-08	4.80E-06
	Sipunculus_f2p32_4655_transcript1020_g1	CANX; calnexin	down	-1.19	0.0005886	0.01132951	up	1.28	0.001635	0.021715
	Sipunculus_f2p45_3116_transcript5211_g1	htpG, HSP90A	down	-2.18	1.11E-05	0.00054434				
	Sipunculus_f6p60_2671_transcript8055_g1	HS90A_RABIT					up	2.07	1.03E-07	8.69E-06
Measles	Sipunculus_f7p60_2477_transcript9537_g1	HSPA1	down	-1.81	4.08E-07	4.00E-05	up	3.41	4.61E-08	4.80E-06
	Sipunculus_f2p28_3135_transcript5416_g1	NFKBIA	down	-1.69	0.00097914	0.0161544	up	1.42	0.001698	0.022183
	Sipunculus_f6p60_3095_transcript5232_g1	EIF2S1	down	-1.58	0.00095804	0.01594029				
FoxO signaling pathway	Sipunculus_f22p60_2506_transcript8785_g1	PRMT1	down	-2.30	1.06E-09	2.60E-07	up	2.18	7.66E-14	4.97E-11
	Sipunculus_f2p56_1933_transcript15246_g1	GADD45	down	-1.91	0.00073695	0.01319348				
	Sipunculus_f3p52_2644_transcript8165_g1	KLF2	down	-2.29	1.98E-06	0.00014373	up	2.56	1.80E-06	0.000102
	Sipunculus_f5p60_3036_transcript5531_g1	MEK1	down	-1.40	1.82E-07	2.03E-05				
	Sipunculus_f2p13_7308_transcript62_g1	PRKAA	up	1.36	4.40E-06	0.00026051	down	-1.51	1.96E-05	0.000744
	Sipunculus_f2p31_5343_transcript550_g1	FBXO25_32	up	2.08	0.00234829	0.03026568	down	-2.42	0.000223	0.004884
	Sipunculus_f2p21_4943_transcript755_g1	PRKAB	up	1.78	2.46E-13	1.81E-10				
Toxoplasmosis	Sipunculus_f7p60_2477_transcript9537_g1	HSPA1	down	-1.81	4.08E-07	4.00E-05	up	3.41	4.61E-08	4.80E-06
	Sipunculus_f2p28_3135_transcript5416_g1	NFKBIA	down	-1.69	0.00097914	0.0161544	up	1.42	0.001698	0.022183

	Sipunculus_f2p11_5185_transcript653_g1	BIRC2_HUMAN					up	1.73	0.000707	0.011611
Estrogen signaling pathway	Sipunculus_f7p60_2477_transcript9537_g1	HSPA1	down	-1.81	4.08E-07	4.00E-05	up	3.41	4.61E-08	4.80E-06
	Sipunculus_f2p45_3116_transcript5211_g1	htpG, HSP90A	down	-2.18	1.11E-05	0.00054434				
	Sipunculus_f2p54_2779_transcript7188_g1	FKBP4_5	down	-1.17	9.73E-05	0.0028381	up	1.30	0.000286	0.005947
	Sipunculus_f5p60_3036_transcript5531_g1	MEK1	down	-1.40	1.82E-07	2.03E-05				
	Sipunculus_f5p56_2163_transcript12903_g	CALL4_BOVIN					up	1.31	0.0009	0.01382
	Sipunculus_f6p60_2671_transcript8055_g1	HS90A_RABIT					up	2.07	1.03E-07	8.69E-06
Longevity regulating pathway - multiple species	Sipunculus_f7p60_2477_transcript9537_g1	HSPA1	down	-1.81	4.08E-07	4.00E-05	up	3.41	4.61E-08	4.80E-06
	Sipunculus_f6p60_3741_transcript2638_g1	CRYAB_CHICK	down	-3.43	0.00012101	0.00339409				
	Sipunculus_f8p60_1918_transcript15288_g1	SODC_PBCV1					up	1.57	0.001347	0.018485
	Sipunculus_f2p13_7308_transcript62_g1	PRKAA	up	1.36	4.40E-06	0.00026051	down	-1.51	1.96E-05	0.000744
	Sipunculus_f2p21_4943_transcript755_g1	PRKAB	up	1.78	2.46E-13	1.81E-10				
	Sipunculus_f28p60_2933_transcript6091_g1	AKTS1_HUMAN					down	-2.05	0.002166	0.02632
	Sipunculus_f7p54_7655_transcript46_g1	ADCY9_MOUSE					down	-1.95	8.44E-05	0.002268
	Sipunculus_f8p60_3054_transcript5592_g1	AKTS1_HUMAN					down	-2.94	2.57E-19	2.99E-16
Aminocayl-tRNA biosynthesis	Sipunculus_f11p60_3036_transcript5533_g1	QARS	down	-1.34	0.000439	0.009097	up	1.33	0.0000632	0.0109
	Sipunculus_f2p13_3265_transcript4250_g1	SARS	down	-1.37	0.001323	0.019876	up	1.17	0.001957	0.02462
	Sipunculus_f2p36_3901_transcript2213_g1	RARS	down	-1.77	5.29E-09	9.44E-07	up	1.17	0.003023	0.03377
	Sipunculus_f4p60_2568_transcript8875_g1	KARS	down	-2.71	2.48E-05	0.000974	up	2.39	9.11E-06	0.000411

Sipunculus_f5p60_3215_transcript4632_g1	TARS	down	-1.52	1.01E-05	0.000511	up	1.37	5.68E-05	0.001734
Sipunculus_f6p60_3110_transcript5288_g1	DARS	down	-1.3778	0.000549	0.01088				

Table S3. The annotated genes in the top 10 pathways from KEGG enrichment analysis.

Gene name	KEGG pathways
HSPA1_8; heat shock 70kDa protein 1/8	Influenza A， Legionellosis，Antigen processing and presentation，Measles，MAPK signaling pathway，Toxoplasmosis，Longevity regulating pathway - multiple species，Estrogen signaling pathway，Protein processing in endoplasmic reticulum，Epstein-Barr virus infection
MAP2K1, MEK1; mitogen-activated protein kinase kinase 1 [EC:2.7.12.2]	Influenza A，MAPK signaling pathway，Melanoma，FoxO signaling pathway， Estrogen signaling pathway，Bladder cancer，Apoptosis，VEGF signaling pathway
NFKBIA; NF-kappa-B inhibitor alpha	Influenza A，Legionellosis，Measles，Toxoplasmosis，NOD-like receptor signaling pathway，Apoptosis，Leishmaniasis，Epstein-Barr virus infection
MAP3K7IP1, TAB1; TAK1-binding protein 1	MAPK signaling pathway，Toxoplasmosis，NOD-like receptor signaling pathway， Leishmaniasis，Epstein-Barr virus infection
CRYAB; crystallin, alpha B	Protein processing in endoplasmic reticulum
HSPB1; heat shock protein beta-1	MAPK signaling pathway，VEGF signaling pathway
CYP2J; cytochrome P450, family 2, subfamily J [EC:1.14.14.1]	Linoleic acid metabolism，Longevity regulating pathway - worm
htpG, HSP90A; molecular chaperone HtpG	Antigen processing and presentation，Estrogen signaling pathway，Protein processing in endoplasmic reticulum
GADD45; growth arrest and DNA-damage-inducible protein	MAPK signaling pathway，Melanoma，Apoptosis，FoxO signaling pathway，
CANX; calnexin	Antigen processing and presentation，Protein processing in endoplasmic reticulum
PRKAB; 5-AMP-activated protein kinase, regulatory beta subunit	FoxO signaling pathway，Longevity regulating pathway - multiple species，Circadian rhythm
PRKAG; 5-AMP-activated protein kinase, regulatory gamma subunit	FoxO signaling pathway，Longevity regulating pathway - multiple species，Circadian rhythm

Of the DEGs, 12 genes (*HSPA1_8*, *MAP2K1* (*MEK1*), *NFKBIA*, *MAP3K7IP1* (*TAB1*), *CRYAB*, *HSPB1*, *CYP2J*, *GADD45*, *HSP90A* (*htpG*), *CANX*, *PRKAB* and *PRKAG*) appeared most frequently in the comparison groups (H vs. M, M vs. L and L vs. H), and they are displayed in Tab. S3.

Table S4. The DEGs and function annotation present in clusters 1, 2 and 7.

	Gene ID	Function annotation
**Cluster 1**	Sipunculus_f3p35_1914_transcript16239_g1	PREDICTED: KH domain-containing, RNA-binding, signal transduction-associated protein 2-like [Leptonychotes weddellii]
	Sipunculus_f2p50_3343_transcript3972_g1	hypothetical protein CAPTEDRAFT_202917 [Capitella teleta]
	Sipunculus_f4p60_1986_transcript14428_g1	PREDICTED: enolase-phosphatase E1 [Latimeria chalumnae]
	Sipunculus_f2p37_4603_transcript1099_g1	hypothetical protein CAPTEDRAFT_163980 [Capitella teleta]

Sipunculus_f6p60_1297_transcript19499_g1	putative FK506-binding protein [Suberites domuncula]
Sipunculus_f3p55_2521_transcript9141_g1	hypothetical protein BRAFLDRAFT_284855 [Branchiostoma floridae]
Sipunculus_f3p27_5380_transcript533_g1	PREDICTED: follistatin-A-like isoform X1 [Lingula anatina]
Sipunculus_f2p59_1507_transcript18724_g1	PREDICTED: activator of 90 kDa heat shock protein ATPase homolog 1-like [Lingula anatina]
Sipunculus_f3p56_2884_transcript6501_g1	hypothetical protein CAPTEDRAFT_156414 [Capitella teleta]
Sipunculus_f10p60_890_transcript20406_g1	hypothetical protein AC249_AIPGENE6048 [Exaiptasia pallida]
Sipunculus_f4p60_1730_transcript17227_g2	PREDICTED: RNA-binding protein with serine-rich domain 1-like [Lingula anatina]
Sipunculus_f6p60_2412_transcript10067_g1	hypothetical protein CAPTEDRAFT_161470 [Capitella teleta]
Sipunculus_f4p60_1545_transcript18354_g1	RecName: Full=Cytochrome c
Sipunculus_f4p60_1082_transcript20150_g1	PREDICTED: 10 kDa heat shock protein, mitochondrial isoform X2 [Latimeria chalumnae]
Sipunculus_f2p60_2710_transcript7692_g1	PREDICTED: 3-oxoacyl-[acyl-carrier-protein] reductase FabG-like isoform X1 [Lingula anatina]
Sipunculus_f3p57_1966_transcript14662_g1	PREDICTED: inositol-3-phosphate synthase 1-A-like [Lingula anatina]
Sipunculus_f2p60_2052_transcript13877_g2	chaperonin containing tcp1 [Haliotis discus discus]
Sipunculus_f4p60_2240_transcript11973_g2	PREDICTED: pancreatic lipase-related protein 2-like [Nasonia vitripennis]
Sipunculus_f2p24_2175_transcript11323_g1	hypothetical protein CAPTEDRAFT_223809 [Capitella teleta]
Sipunculus_f3p35_4276_transcript1514_g1	hypothetical protein CAPTEDRAFT_160144 [Capitella teleta]
Sipunculus_f4p54_1868_transcript15532_g1	hypothetical protein LOTGIDRAFT_157797 [Lottia gigantea]
Sipunculus_f8p60_5106_transcript700_g1	PREDICTED: microphthalmia-associated transcription factor-like isoform X3 [Crassostrea gigas]
Sipunculus_f3p60_767_transcript20585_g2	hypothetical protein CGI_10000492 [Crassostrea gigas]
Sipunculus_f10p60_3062_transcript5497_g1	hypothetical protein CAPTEDRAFT_224386 [Capitella teleta]
Sipunculus_f2p13_3265_transcript4250_g1	PREDICTED: serine--tRNA ligase, cytoplasmic-like [Aplysia californica]
Sipunculus_f3p48_2128_transcript13054_g1	PREDICTED: multifunctional protein ADE2-like [Crassostrea gigas]
Sipunculus_f2p34_3496_transcript3382_g1	hypothetical protein LOTGIDRAFT_130231, partial [Lottia gigantea]
Sipunculus_f6p60_4276_transcript1495_g1	hypothetical protein CAPTEDRAFT_134807, partial [Capitella teleta]
Sipunculus_f5p45_3000_transcript5676_g1	PREDICTED: ATP-binding cassette sub-family E member 1-like [Aplysia californica]
Sipunculus_f2p53_2306_transcript11202_g1	PREDICTED: aladin-like [Lingula anatina]
Sipunculus_f22p60_2506_transcript8785_g1	PREDICTED: protein arginine N-methyltransferase 1-like [Octopus bimaculoides]
Sipunculus_f2p52_2601_transcript8604_g2	PREDICTED: uncharacterized protein LOC106157175 [Lingula anatina]
Sipunculus_f2p45_2456_transcript9715_g1	PREDICTED: T-complex protein 1 subunit zeta-like [Lingula anatina]
Sipunculus_f3p52_2644_transcript8165_g1	kruppel-like factor 1/2/4 [Leptochiton asellus]
Sipunculus_f2p20_3244_transcript4482_g1	PREDICTED: uncharacterized protein LOC105343207 isoform X4 [Crassostrea gigas]
Sipunculus_f2p29_2983_transcript5977_g1	PREDICTED: chymotrypsinogen A-like [Lingula anatina]
Sipunculus_f2p54_2779_transcript7188_g1	hypothetical protein LOTGIDRAFT_102242 [Lottia gigantea]

Sipunculus_f21p60_2267_transcript11731_g1	PREDICTED: dolichyl-diphosphooligosaccharide--protein glycosyltransferase subunit 2-like isoform X3 [Aplysia californica]
Sipunculus_f2p42_3913_transcript2193_g1	hypothetical protein CAPTEDRAFT_229004 [Capitella teleta]
Sipunculus_f3p60_3685_transcript2783_g2	hypothetical protein CAPTEDRAFT_181870 [Capitella teleta]
Sipunculus_f2p15_3326_transcript4212_g1	hypothetical protein LOTGIDRAFT_204438 [Lottia gigantea]
Sipunculus_f5p60_3215_transcript4632_g1	hypothetical protein CAPTEDRAFT_158346 [Capitella teleta]
Sipunculus_f4p46_3102_transcript5057_g1	PREDICTED: cytosolic non-specific dipeptidase-like [Maylandia zebra]
Sipunculus_f9p60_2011_transcript13559_g1	PREDICTED: uncharacterized protein LOC106153767 [Lingula anatina]
Sipunculus_f2p45_3174_transcript4743_g1	PREDICTED: heterogeneous nuclear ribonucleoprotein R-like isoform X7 [Harpegnathos saltator]
Sipunculus_f2p34_1933_transcript15070_g1	PREDICTED: spliceosome RNA helicase DDX39B [Crassostrea gigas]
Sipunculus_f2p34_3140_transcript4953_g1	hypothetical protein CAPTEDRAFT_221834 [Capitella teleta]
Sipunculus_f2p60_1878_transcript15638_g1	hypothetical protein CAPTEDRAFT_215353 [Capitella teleta]
Sipunculus_f10p60_2747_transcript7660_g1	hypothetical protein CAPTEDRAFT_181489 [Capitella teleta]
Sipunculus_f13p60_3004_transcript5726_g1	PREDICTED: matrix metalloproteinase-16-like [Lingula anatina]
Sipunculus_f27p60_1856_transcript16022_g1	PREDICTED: uncharacterized protein LOC105667360 [Linepithema humile]
Sipunculus_f7p60_3280_transcript4274_g1	PREDICTED: trifunctional enzyme subunit alpha, mitochondrial-like [Biomphalaria glabrata]
Sipunculus_f3p57_2394_transcript10474_g1	PREDICTED: uncharacterized protein LOC106868732 isoform X1 [Octopus bimaculoides]
Sipunculus_f4p60_1717_transcript17323_g2	PREDICTED: leukocyte antigen CD37 isoform X1 [Camelus ferus]
Sipunculus_f3p60_2203_transcript12309_g1	PREDICTED: T-complex protein 1 subunit eta-like isoform X1 [Lingula anatina]
Sipunculus_f11p60_3036_transcript5533_g1	PREDICTED: glutamine--tRNA ligase-like [Lingula anatina]
Sipunculus_f5p60_3695_transcript2696_g1	PREDICTED: mitochondrial 2-oxoglutarate/malate carrier protein-like [Limulus polyphemus]
Sipunculus_f2p39_2761_transcript7227_g1	hypothetical protein LOTGIDRAFT_128909, partial [Lottia gigantea]
Sipunculus_f8p60_2689_transcript7798_g1	PREDICTED: stress-induced-phosphoprotein 1-like [Lingula anatina]
Sipunculus_f3p60_1145_transcript19952_g4	PREDICTED: small nuclear ribonucleoprotein F [Lingula anatina]
Sipunculus_f11p60_2784_transcript6547_g1	chaperonin subunit, putative [Ixodes scapularis]
Sipunculus_f2p50_2563_transcript8840_g1	PREDICTED: myelin expression factor 2-like isoform X2 [Octopus bimaculoides]
Sipunculus_f2p32_4655_transcript1020_g1	hypothetical protein CAPTEDRAFT_170444 [Capitella teleta]
Sipunculus_f3p43_3129_transcript5014_g1	PREDICTED: coiled-coil domain-containing protein 173-like [Lingula anatina]
Sipunculus_f2p22_4187_transcript1643_g1	PREDICTED: MAM and LDL-receptor class A domain-containing protein 1-like [Lingula anatina]
Sipunculus_f8p60_2886_transcript6545_g1	hypothetical protein LOTGIDRAFT_200177 [Lottia gigantea]
Sipunculus_f6p60_1939_transcript15139_g2	elongation factor 1-alpha [Danio rerio]

	Sipunculus_f18p60_3559_transcript3151_g1	PREDICTED: proliferation-associated protein 2G4-like isoform X1 [Saccoglossus kowalevskii]
	Sipunculus_f2p7_1874_transcript16074_g1	PREDICTED: RNA-binding protein lark-like [Crassostrea gigas]
	Sipunculus_f2p60_1331_transcript19407_g1	PREDICTED: ubiquitin-fold modifier-conjugating enzyme 1 [Crassostrea gigas]
**Cluster 2**	Sipunculus_f2p60_2550_transcript8974_g2	PREDICTED: TOX high mobility group box family member 4-A-like [Limulus polyphemus]
	Sipunculus_f2p42_2764_transcript7239_g1	PREDICTED: hemicentin-1-like [Halyomorpha halys]
	Sipunculus_f2p52_2557_transcript8894_g1	heat shock protein 70 [Cellana toreuma]
	Sipunculus_f7p60_2700_transcript7816_g2	GPI-linked carbonic anydrase [Cherax quadricarinatus]
	Sipunculus_f4p60_2257_transcript11671_g1	PREDICTED: stress-70 protein, mitochondrial [Gekko japonicus]
	Sipunculus_f2p21_3803_transcript2461_g1	hypothetical protein CAPTEDRAFT_223748 [Capitella teleta]
	Sipunculus_f25p60_1530_transcript18479_g1	cofilin [Sphaeroforma arctica JP610]
	Sipunculus_f2p60_1702_transcript17384_g3	Tubulin alpha-1 chain [Acromyrmex echinatior]
	Sipunculus_f7p60_2477_transcript9537_g1	heat shock protein 70 [Rhagoletis pomonella]
	Sipunculus_f2p26_2337_transcript11013_g1	PREDICTED: protein Skeletor, isoforms B/C-like [Lingula anatina]
	Sipunculus_f2p36_4465_transcript1232_g1	hypothetical protein CAPTEDRAFT_228725 [Capitella teleta]
	Sipunculus_f4p60_538_transcript20798_g1	hypothetical protein BRAFLDRAFT_152201 [Branchiostoma floridae]
	Sipunculus_f3p60_2899_transcript6438_g2	hypothetical protein HELRODRAFT_175309 [Helobdella robusta]
	Sipunculus_f2p13_2622_transcript8499_g1	heat shock protein 70 [Rhagoletis pomonella]
**Cluster 7**	Sipunculus_f2p29_2771_transcript7191_g1	PREDICTED: LOW QUALITY PROTEIN: protein toll-like [Lingula anatina]
	Sipunculus_f3p48_4847_transcript848_g1	hypothetical protein HELRODRAFT_110263 [Helobdella robusta]
	Sipunculus_f2p19_3265_transcript4365_g1	PREDICTED: mid1-interacting protein 1-B-like [Lingula anatina]
	Sipunculus_f3p60_2097_transcript13597_g4	PREDICTED: aldehyde dehydrogenase family 8 member A1 [Pelodiscus sinensis]
	Sipunculus_f3p60_2548_transcript8940_g1	PREDICTED: midnolin-B-like isoform X1 [Halyomorpha halys]
	Sipunculus_f3p20_2734_transcript7600_g1	PREDICTED: zonadhesin-like, partial [Phaethon lepturus]
	Sipunculus_f4p60_2568_transcript8875_g1	hypothetical protein BRAFLDRAFT_276665 [Branchiostoma floridae]
	Sipunculus_f2p15_3742_transcript2617_g1	PREDICTED: signal peptide, CUB and EGF-like domain-containing protein 3 [Lingula anatina]
	Sipunculus_f2p30_1777_transcript16625_g1	RecName: Full=Hemerythrin
	Sipunculus_f2p54_2646_transcript8139_g1	PREDICTED: uncharacterized protein LOC106176979 isoform X3 [Lingula anatina]
	Sipunculus_f2p37_2999_transcript5830_g1	PREDICTED: unconventional myosin-VI-like [Lingula anatina]
	Sipunculus_f4p60_3148_transcript4891_g2	PREDICTED: uncharacterized protein LOC100892120 [Strongylocentrotus purpuratus]
	Sipunculus_f9p60_4425_transcript1317_g1	PREDICTED: inorganic pyrophosphatase-like [Saccoglossus kowalevskii]
	Sipunculus_f5p60_1304_transcript19475_g1	PREDICTED: ras-like GTP-binding protein Rho1 isoform X2 [Linepithema humile]
	Sipunculus_f2p60_2154_transcript12808_g3	hypothetical protein CAPTEDRAFT_168933 [Capitella teleta]

Sipunculus_f5p60_3345_transcript4011_g3	PREDICTED: uncharacterized protein LOC106065023 [Biomphalaria glabrata]
Sipunculus_f2p52_3240_transcript4435_g1	hypothetical protein CAPTEDRAFT_223627 [Capitella teleta]
Sipunculus_f3p24_4593_transcript1090_g1	PREDICTED: SCO-spondin-like isoform X4 [Lingula anatina]
Sipunculus_f2p38_3075_transcript5310_g1	hypothetical protein CAPTEDRAFT_164667 [Capitella teleta]
Sipunculus_f2p28_2972_transcript6021_g1	PREDICTED: protein Skeletor, isoforms B/C-like [Lingula anatina]
Sipunculus_f3p36_4238_transcript1577_g1	hypothetical protein LOTGIDRAFT_231476 [Lottia gigantea]
Sipunculus_f2p60_1623_transcript17865_g1	hypothetical protein EAG_16421, partial [Camponotus floridanus]
Sipunculus_f7p60_1827_transcript16193_g1	PREDICTED: syncollin [Esox lucius]
Sipunculus_f2p36_2575_transcript8719_g1	PREDICTED: low-density lipoprotein receptor-related protein 8-like [Halyomorpha halys]
Sipunculus_f3p60_2909_transcript6449_g3	PREDICTED: cytochrome P450 3A8-like [Saccoglossus kowalevskii]
Sipunculus_f4p53_4894_transcript845_g1	PREDICTED: sushi, von Willebrand factor type A, EGF and pentraxin domain-containing protein 1-like [Lingula anatina]
Sipunculus_f5p60_2227_transcript12044_g1	PREDICTED: heterogeneous nuclear ribonucleoprotein R isoform X9 [Linepithema humile]
Sipunculus_f2p28_3135_transcript5416_g1	inhibitor of nuclear factor-kappaB protein [Pinctada fucata]
Sipunculus_f2p24_1685_transcript17407_g1	hypothetical protein HELRODRAFT_187046 [Helobdella robusta]
Sipunculus_f13p60_2933_transcript6302_g1	hypothetical protein CAPTEDRAFT_179168 [Capitella teleta]
Sipunculus_f3p60_1548_transcript18363_g1	ADP ribosylation factor 79F [Argas monolakensis]
Sipunculus_f2p21_1604_transcript17980_g1	PREDICTED: vascular endothelial growth factor A-like [Limulus polyphemus]
Sipunculus_f2p19_3158_transcript4822_g1	PREDICTED: uncharacterized protein LOC100378011 [Saccoglossus kowalevskii]
